# Point-of-Care Ultrasound Use in Hemodynamic Assessment

**DOI:** 10.3390/biomedicines13061426

**Published:** 2025-06-10

**Authors:** Ahmed Noor, Margaret Liu, Alan Jarman, Travis Yamanaka, Malvika Kaul

**Affiliations:** 1 Department of Medicine, University of Illinois at Chicago, Chicago, IL 60612, USA; 2 Division of Academic Internal Medicine, University of Illinois at Chicago, Chicago, IL 60612, USA; 3 Pulmonary, Critical Care, and Sleep Medicine, Jesse Brown Veterans Affairs Medical Center, Chicago, IL 60612, USA; 4 Division of Pulmonary, Critical Care, Sleep, and Allergy, Department of Medicine, University of Illinois at Chicago, Chicago, IL 60612, USA

**Keywords:** ultrasound, point of care, critical care, emergency medicine, volume assessment, echocardiography, hemodynamics, cardiac arrest

## Abstract

Hemodynamic assessment is critical in emergency and critical care for preventing, diagnosing, and managing shock states that significantly affect patient outcomes. Point-of-care ultrasound (POCUS) has become an invaluable, non-invasive, real-time, and reproducible tool for bedside decision-making. Advancements such as Doppler imaging, advanced critical care ultrasonography, and transesophageal echocardiography (TEE) have expanded its utility, enabling rapid and repeatable evaluations, especially in complex mixed shock presentations. This review explores the role of POCUS in hemodynamic monitoring, emphasizing its ability to assess cardiac output, filling pressures, and vascular congestion, facilitating shock classification and guiding fluid management. We highlight an extensive array of POCUS techniques for evaluating right and left cardiac function and review existing literature on their advantages, limitations, and appropriate clinical applications. Beyond assessing volume status, this review discusses the role of POCUS in predicting fluid responsiveness and supporting more individualized, precise management strategies. Ultimately, while POCUS is a powerful tool for rapid, comprehensive hemodynamic assessment in acute settings, its limitations must be acknowledged and thoughtfully integrated into clinical decision-making.

## 1. Introduction

Effective hemodynamics depends on a balance between the demand, delivery, and extraction of oxygen to maintain optimal perfusion and cellular function. This balance relies on a functional circulatory system, and its disruption can lead to shock [1]. Circulatory failure is thought to result from a loss of coherence between macrocirculation (heart and large vessels) and microcirculation (arterioles, venules, and capillaries) [2,3,4].

Traditionally, shock management focuses on optimizing macro circulation through fluid resuscitation and vasoactive agents to enhance cardiac output and tissue perfusion. However, this approach may overlook microcirculatory dysfunction where vascular, hormonal, biochemical, and neural factors may impair perfusion despite macrocirculatory stabilization. Over-resuscitation increases the risk of volume overload and patient harm [5]. Defining optimal fluid resuscitation remains challenging, as direct microcirculatory assessment is not yet clinically available. Techniques such as near-infrared spectroscopy of the sublingual mucosa have been described but are not widely implemented [6]. Refining systemic hemodynamic monitoring is crucial for guiding resuscitative efforts.

Shock states arise from decreased cardiac output (CO) and subsequent inadequate systemic perfusion due to one of four mechanisms: reduced circulating volume (hypovolemic shock), impaired cardiac function (cardiogenic shock), obstruction of blood flow (obstructive shock), or abnormal blood flow distribution leading to perfusion failure (distributive shock) (Table 1). Effective management requires an understanding of oxygen delivery (DO_2_), the primary target in resuscitation. DO_2_ depends on the CO and arterial oxygen content (CaO_2_), as represented by the oxygen delivery equation:

(1) $${DO}_{2}=CO\times \left[\left(Hgb\times 1.34\times {SaO}_{2}\right)+\left({PaO}_{2}\times 0.003\right)\right]$$

(Hgb: hemoglobin concentration, SaO_2_: arterial oxygen saturation, and PaO_2_: partial pressure of oxygen in arterial blood.)

Thus, resuscitation efforts focus on optimizing these components to restore adequate oxygen delivery.

Hemodynamic monitoring parameters in shock states are traditionally categorized as static or dynamic. Static parameters, such as central venous pressure (CVP) and pulmonary artery occlusion pressure (PAOP), provide only a snapshot of the circulatory system, and though these parameters have been historically helpful for suggesting a course of action for fluid management, their limitations as static measures have prompted the exploration of alternative methods of hemodynamic monitoring. As a result, dynamic parameters, such as stroke volume or pulse pressure variation, have gained more attention for their ability to assess hemodynamic responses to interventions or physiological changes, making them better predictors of fluid responsiveness. Consequently, the Surviving Sepsis Guidelines recommend using dynamic measures over static ones to guide fluid resuscitation [8].

Frontline providers require real-time decision-making tools to guide hemodynamic management. Various monitoring systems exist, ranging from invasive to non-invasive, with varying levels of supporting evidence. Pulmonary artery catheters (PACs) were once considered routine for hemodynamic assessment but are not readily used in practice due to their invasiveness, complexity, and lack of demonstrated benefit in sepsis, partly due to inter-operator variability in interpretation [9].

An ideal monitoring device should provide real-time feedback, be easy to use, diagnostically accurate, and be minimally or non-invasive. While no single device fully meets these criteria, point-of-care ultrasound (POCUS) has emerged as a promising tool. In addition to offering real-time imaging, ease of use, and practicality in clinical decision-making, POCUS is non-invasive and highly reproducible, and its measurements are increasingly standardized across clinical investigations. These qualities make it an attractive option for hemodynamic assessment in shock states.

This review explores the role of ultrasound in hemodynamic monitoring, highlighting its potential as a user-friendly tool that provides real-time feedback for managing circulatory failure.

### Literature Search Methodology

A targeted literature search was conducted using PubMed and Google Scholar from January 2000 to April 2025. Search terms included a combination of “point-of-care ultrasound”, “POCUS”, “hemodynamic assessment”, “shock”, and “fluid responsiveness”. Relevant peer-reviewed articles, guidelines, and reviews in English were included. Titles and abstracts were screened for relevance, followed by the full text review. The reference list of the key articles was also reviewed to identify additional pertinent studies.

## 2. Critical Care Ultrasonography

Ultrasound has been used in clinical practice since the early 1980s [10]. With the advent of portable devices, its use has become increasingly popular among emergency and critical care providers for routine bedside applications. While proper interpretations and usage require training, standardized POCUS education is increasingly being integrated into medical curricula, further enhancing its value as a diagnostic tool [7].

Ultrasound generates sound waves to visualize internal structures, primarily in two-dimensional modes such as B-mode (brightness mode). Additional applications include M-mode (motion mode) and the various Doppler modes, which enable more specialized assessments [11]. Critical care ultrasonography refers to POCUS performed on critically ill patients, combining cardiac and large vessel imaging with ultrasound of the lungs, pleural cavity, abdomen, and lower extremities. Dynamic measurements obtained through POCUS, such as cardiac output (CO), can aid real-time therapeutic decision-making and have been shown to be comparable to PACs [12]. Furthermore, beyond basic views, additional training in advanced critical care echocardiographic (ACCE) techniques and the introduction of transesophageal echocardiography (TEE) provide valuable insights into right and left ventricular function (RV and LV) and support more detailed hemodynamic assessments [13].

**Figure 1:**
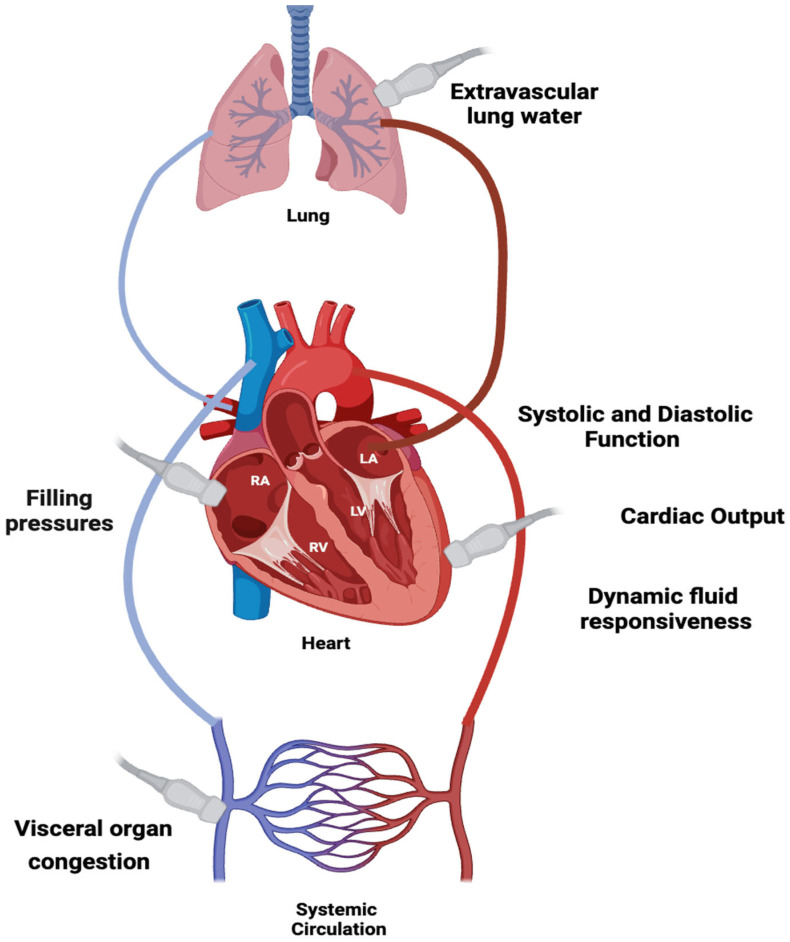
Ultrasound integration in hemodynamic assessment evaluating cardiac output, fluid responsiveness, filling pressures, extravascular lung water, and visceral congestion.

Integrating bedside ultrasound into hemodynamic monitoring allows clinicians to assess the key components of oxygen delivery, identifying which factors are impaired in various shock states. A central focus is the assessment of cardiac output (CO), primarily determined by stroke volume, which depends on preload, contractility, and afterload. Ultrasound facilitates the dynamic evaluation of these parameters, guiding resuscitation efforts and therapy adjustments. In particular, it aids in assessing fluid responsiveness, monitoring the effects of inotropic or vasopressor therapy, and detecting complications such as venous congestion and extravascular lung water accumulation (Figure 1). This review will explore ultrasound-based techniques for evaluating both right and left ventricular function, with special attention to fluid responsiveness assessments and the role of transesophageal echocardiography (TEE) in critically ill patients.

## 3. Right Ventricular Assessment and Integration of Ultrasound

### 3.1. Right Ventricular Preload

Preload refers to the ventricular end-diastolic volume or pressure, which, in the right ventricle (RV), reflects systemic venous return. The RV, being more compliant than the left ventricle (LV), can tolerate sudden increases in preload [14]. However, a chronic elevation in preload can overwhelm RV compliance, leading to venous congestion, peripheral edema, congestive hepatopathy, heart failure, and acute kidney injury (AKI) [15,16]. Conversely, low preload, often from volume depletion or decreased systemic vascular resistance, also carries clinical risk [14]. Assessing RV preload is essential in guiding fluid management, with CVP serving as a key measurement. It reflects right atrial pressures (RAP), provided that the inferior vena cava (IVC) is contiguous with the right atrium.

**Figure 2:**
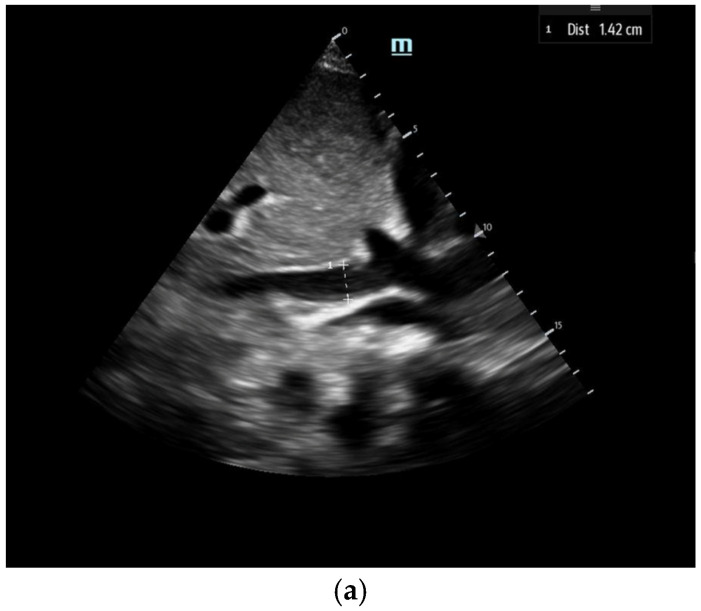

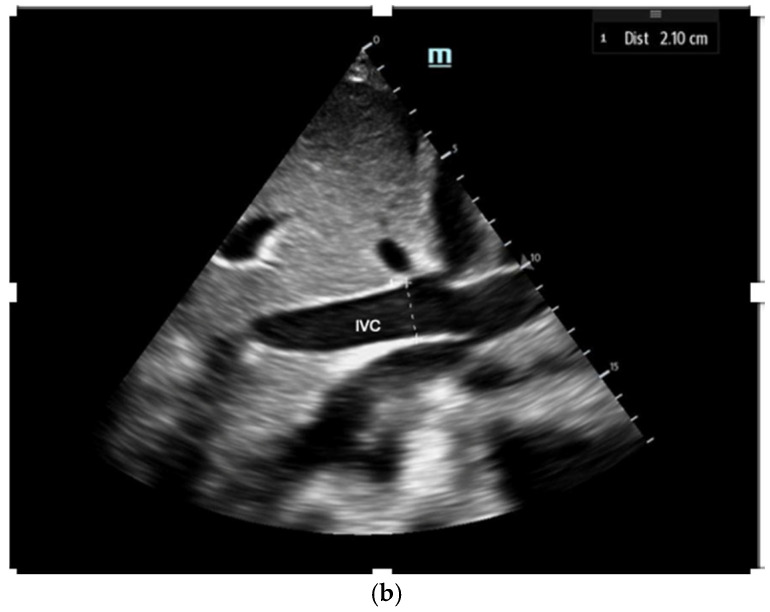
The IVC (Inferior Vena Cava) diameter measured in subcoastal view approximately 1 cm below IVC–hepatic vein junction in a spontaneously breathing person during both inspiration (**a**) and expiration (**b**).

Point-of-care ultrasound assessment of RV preload:

1. Inferior vena cava assessment: The ultrasound assessment of IVC size and collapsibility offers a non-invasive method to evaluate CVP and RAP (Figure 2). The IVC’s diameter varies with CVP and intravascular volume, although factors like positioning, respiratory variation, and intrathoracic pressure may also influence IVC size [17]. Studies show a strong correlation between maximum IVC diameter (IVC_max_) and invasive CVP measurement, with the 2018 ASE guidelines recommending measuring IVC_max_ during expiration [17]. The IVC compressibility index (IVCCI), which is a dynamic hemodynamic measurement incorporating maximum and minimum IVC diameter (taken at expiration and inspiration, respectively), also has a strong negative correlation with CVP (Figure 2)

(2) $$IVCCI=\frac{IVCmax-IVCmin}{IVCmax}$$

However, these correlations are weaker in patients receiving positive-pressure ventilation [17]. The exception to this pattern was observed in two studies that studied ventilated patients without positive end-expiratory pressure applied [17].

The ability of the IVCCI to predict fluid responsiveness is variable. A meta-analysis reported sensitivity ranging from 0.39 to 0.86 and specificity from 0.28 to 0.91, with an area under the curve (AUC) of 0.71 [18]. However, this meta-analysis highlighted challenges such as significant heterogeneity among the studied populations, the absence of a definitive gold standard for the measurement of fluid responsiveness, and variability in operator technique. Another meta-analysis found an overall pooled sensitivity of 0.63 and a specificity of 0.73 [19]. Additional evidence suggests that only extreme IVCCI values (≥40%) are likely to be useful for predicting fluid responsiveness [20]. Interestingly, although the IVCCI has a weaker correlation to CVP/RAP values in positive-pressure ventilated patients, a different meta-analysis comparing the IVCCI’s ability to specifically predict fluid responsiveness in spontaneous breathing versus positive-pressure ventilated patients found comparable accuracy between both patient populations [21].

2. Internal jugular vein assessment: Similar to IVC ultrasound, the internal jugular vein (IJV) diameter and its distensibility are used to assess fluid responsiveness. In patients on positive-pressure ventilation, the IJV distends inspiration and collapses with expiration. The IJV distensibility index is calculated by comparing the IJV diameter during inspiration and expiration:

(3) $$IJV\;distensibility\;index\;\left(\%\right)=\left(IJVinspiratory-IJVexpiratory\right)\times 100$$

In mechanically ventilated patients on positive-pressure ventilation, fluid responders exhibited significantly higher IJV distensibility before volume expansion and decreased distensibility after a volume challenge, suggesting the utility of IJV distensibility in predicting fluid responsiveness in patients on positive-pressure ventilation [22]. An IJV distensibility index greater than 18% predicted a change in cardiac index of ≥15%, with a sensitivity of 80%, specificity of 95%, and an AUC of 0.915 [22]. Another meta-analysis reported comparable accuracy between the caval and jugular indices in measuring fluid responsiveness [21].

**Figure 3:**
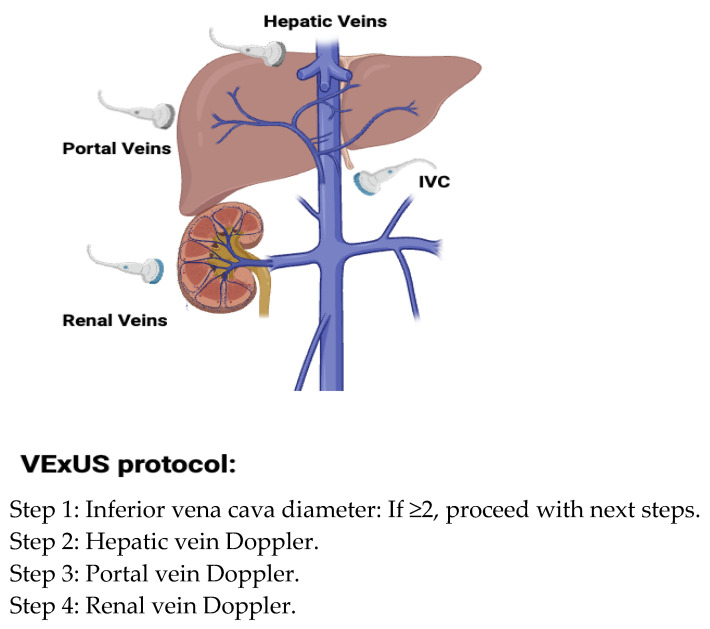
Venous congestion evaluation using the ultrasound (VExUS) protocol for ultrasound scanning [24].

**Figure 4:**
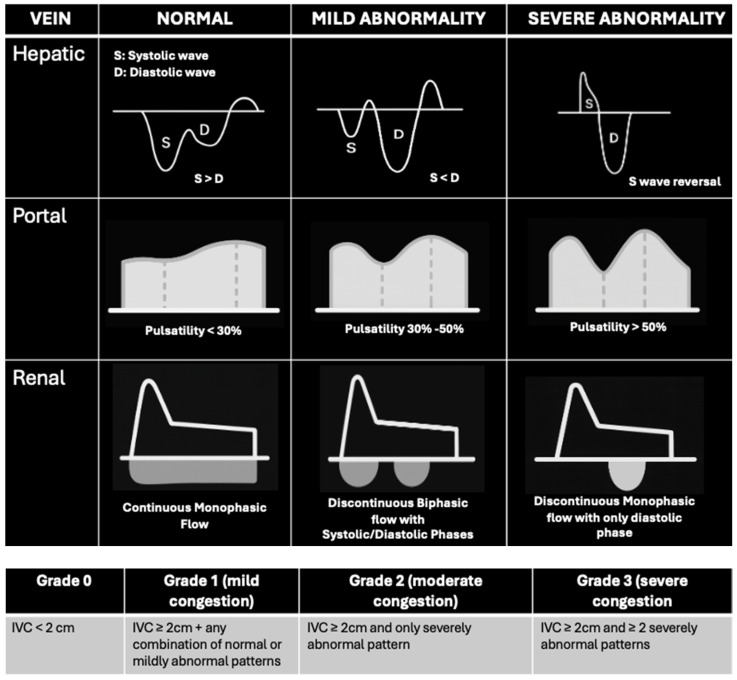
Diagrammatic representation of VExUS patterns: normal and abnormal patterns and grading for congestion.

3. Venous excess ultrasound (VExUS): This has emerged as a valuable adjunct to IVC measurements in evaluating volume-overloaded states, utilizing pulse-wave (PW) Doppler to assess hepatic, portal, and intrarenal venous flow [23] (Figure 3). This technique provides direct insight into peripheral venous congestion and organ dysfunction, with a scoring that incorporates the IVC diameter to grade congestion severity [16] (Figure 4).

Pulse-wave Doppler identifies flow moving towards the transducer and positive deflections and flow moving away as negative, with the amplitude representing speed [16]. Normal venous flow varies by location and is influenced by right atrial pressure (RAP), venous compliance, and distance from the heart. Hepatic vein flow is pulsatile, reflecting RAP variations during systole and diastole due to its direct connection with the IVC. In contrast, flow in more distal intrarenal veins is continuous and no longer reflects RAP [16]. The portal vein, which reflects the portal circulation, also exhibits continuous flow unaffected by RAP. As the RAP increases, pressure changes are transmitted to these peripheral organ veins, altering flow and impacting organ function.

While VExUS is a relatively new hemodynamic tool, studies suggest it correlates with organ dysfunction and disease severity. In cardiac surgery patients, a Grade 3 VExUS score strongly predicted AKI and outperformed CVP in this regard [24]. However, follow-up studies in general ICU patients found no significant association between VExUS and new or worsening AKI, though Grade 2–3 VExUS scores were linked to increased mortality in those with existing AKI [25,26]. One study showed that cardiac surgery patients with a VExUS score of 2 or higher had 4.3 times higher odds of developing postoperative AKI (OR 4.3, 95% CI 1.2–20.7) [27]. In acute decompensated heart failure, a Grade 3 VExUS score was associated with higher 100-day mortality, early readmissions, and worse renal function [28].

While data on VExUS’s guiding fluid management are limited, score reduction after diuretic use was associated with more renal replacement therapy-free days over 28 days [29]. In cardiorenal syndrome, VExUS use improved decongestion and lowered brain natriuretic peptide levels, though without renal function improvement [30]. Like other venous assessments, VExUS cannot differentiate volume from pressure overload (e.g., in obstructive shock). Despite the need for more multicenter data, VExUS shows the prognostic value for systemic congestion, with feasibility rates of 95–100% in critically ill patients when the imaging windows are adequate [27,31].

### 3.2. Right Ventricular Contractility

**Figure 5:**
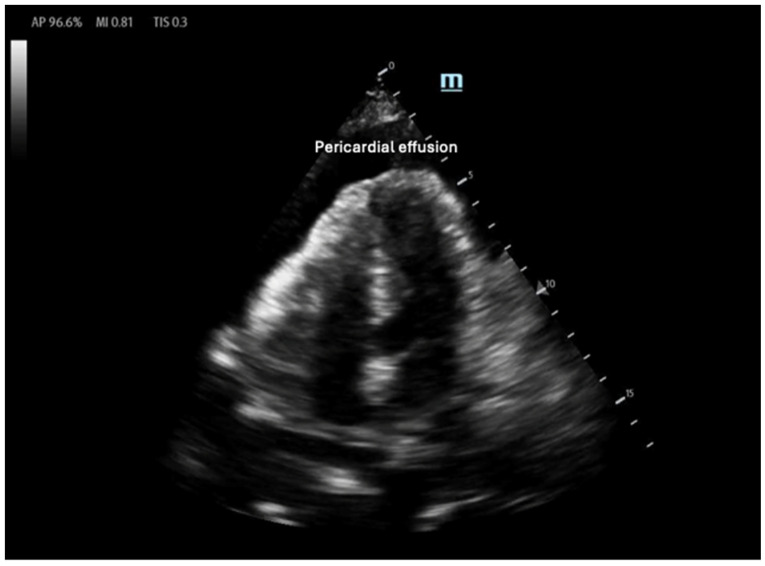
Anechoic pericardial effusion in apical 4-chamber view.

Right ventricular (RV) contractility is essential for maintaining right-sided cardiac output, ensuring blood supply to the pulmonary vasculature and subsequently to the left heart. Unlike the LV, the RV contracts primarily through longitudinal muscle fiber shortening, inward free wall movement, and LV-dependent contraction [32]. As a result, RV contraction occurs predominantly in a longitudinal rather than radial manner. Due to the RV’s greater compliance compared with the LV, its contractility and CO are highly influenced by changes in afterload, which will be discussed in the next section. External pressure changes, such as those seen in cardiac tamponade, can also significantly impact RV function. In tamponade, pericardial fluid compresses the heart chamber, elevating RV diastolic filling pressure and reducing RV output [33]. Owing to the RV’s increased compliance, the RA and the RV are the first chambers to collapse under increased pericardial pressure. Therefore, RA/RV collapse on bedside POCUS in the presence of a pericardial effusion should raise the suspicion of tamponade (Figure 5) [34].

**Figure 6:**
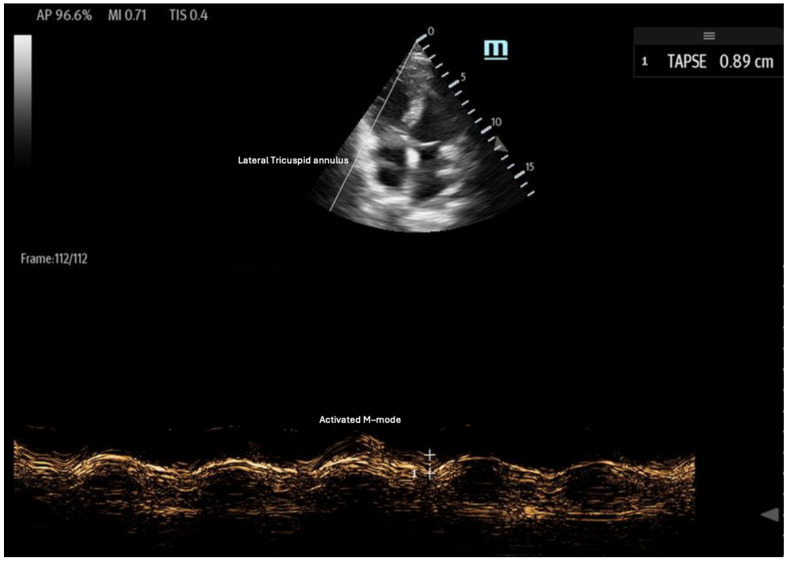
Tricuspid annular plane systolic excursion (TAPSE): apical 4-chamber view with M-mode activation, demonstrating reduced TAPSE (measured at the bottom 2D M-mode).

**Figure 7:**
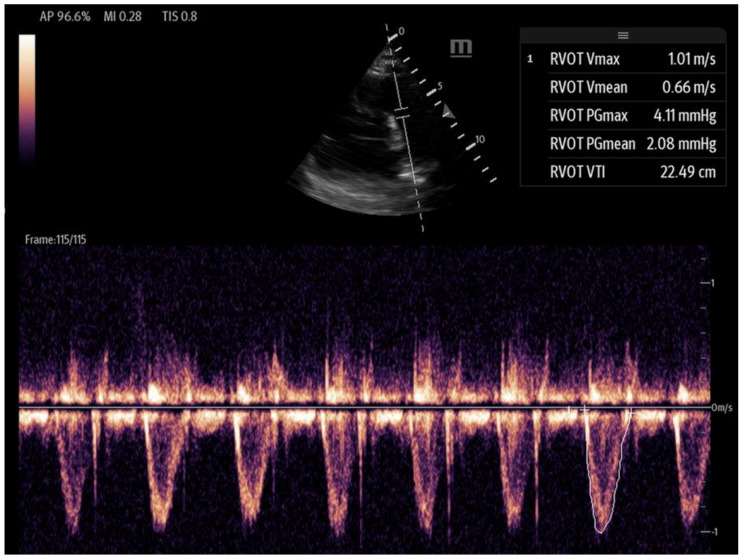
A right ventricular tract VTI in a modified parasternal short axis view, activating the PW doppler.

Point-of-care echocardiographic parameters used to assess RV contractility:

1. Tricuspid annular plane systolic excursion (TAPSE): TAPSE is a simple and widely used measure of RV systolic function, assessed using the M-mode of the 2D ultrasound in the apical four-chamber view (Figure 6). It quantifies the displacement of the tricuspid lateral annulus towards the cardiac apex during systole, reflecting the RV’s longitudinal contraction [35]. Greater displacement indicates better RV contractility, while a TAPSE < 17 mm is indicative of RV systolic dysfunction, with high specificity and low sensitivity [35,36,37]. TAPSE is advantageous due to its ease of measurement; however, it is limited by its two-dimensional (2D) interpretation of a 3D structure. Additionally, its accuracy can be affected by ultrasound probe positioning and increased RV afterload [36].
2. Right ventricular outflow tract velocity–time integral (RVOT-VTI): RV contractility can be evaluated using the RVOT-VTI, which represents the distance traveled by blood across the RVOT during systole. Stroke volume is calculated by multiplying the RVOT cross-sectional area by the RVOT-VTI. This measurement provides insight into both RV function and pulmonary vascular resistance, with an RVOT-VTI ≥ 12 cm considered normal [38] (Figure 7). The RVOT VTI is a better predictor of RV dysfunction in cardiogenic shock states compared to TAPSE and may also have prognostic value in patients with pulmonary hypertension [39,40].
3. Peak lateral tricuspid annular systolic velocity (S′ or systolic wave prime): S′ can be calculated by activating tissue Doppler imaging (TDI) at the lateral tricuspid annulus in the apical four-chamber view. An S′ velocity of less than 10 cm/s correlates strongly with RV systolic dysfunction and may even correlate better than TAPSE for normal RVEF to mild degrees of RVEF reduction. Specifically, an S′ < 11.5 cm/s correlates with an RVEF < 45%, with a sensitivity of 90% and a specificity of 85% [41]. The advantages of S′ include its ease and simplicity; however, similar to TAPSE, it is also limited by its one-dimensionality, as well as its load- and angle-dependence [36,42]. S′ alone is insufficient to capture regional wall motion abnormalities [36].

To provide practical context to bedside use, we summarized the normal values and reported diagnostic accuracy of these right ventricular function parameters in Table 2.

### 3.3. Right Ventricular Afterload

Right ventricular afterload is defined as the pressure that the RV must overcome during systolic ejection [32]. It is largely influenced by pulmonary vascular resistance (PVR), assessing pulmonary artery (PA) pressure (or its surrogates), a key component of RV afterload evaluation [43]. In patients with shock and heart failure, understanding the degree of pulmonary resistance and hypertension is critical for guiding fluid management. PA pressures often rise with increased left-sided pressures and congestion and decrease with fluid decongestion; however, intrinsic pulmonary hypertension should always be considered a potential confounder [35,43].

**Figure 8:**
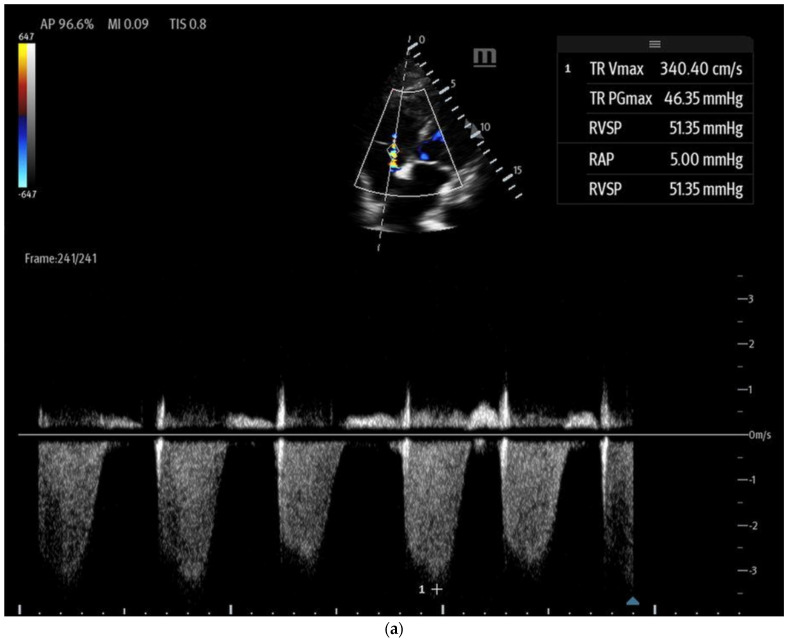

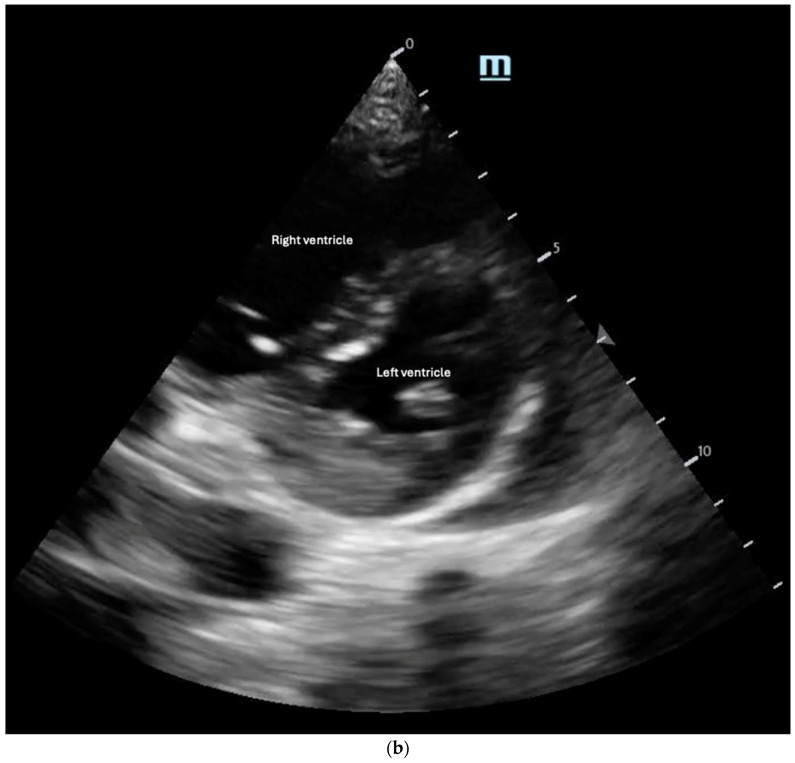
(**a**) Tricuspid regurgitation velocity (TRV) measured by continuous wave (CW) Doppler in apical 4-chamber view. (**b**) D sign in parasternal short axis view in diastolic frame. Note flattening of the interventricular septum.

**Figure 9:**
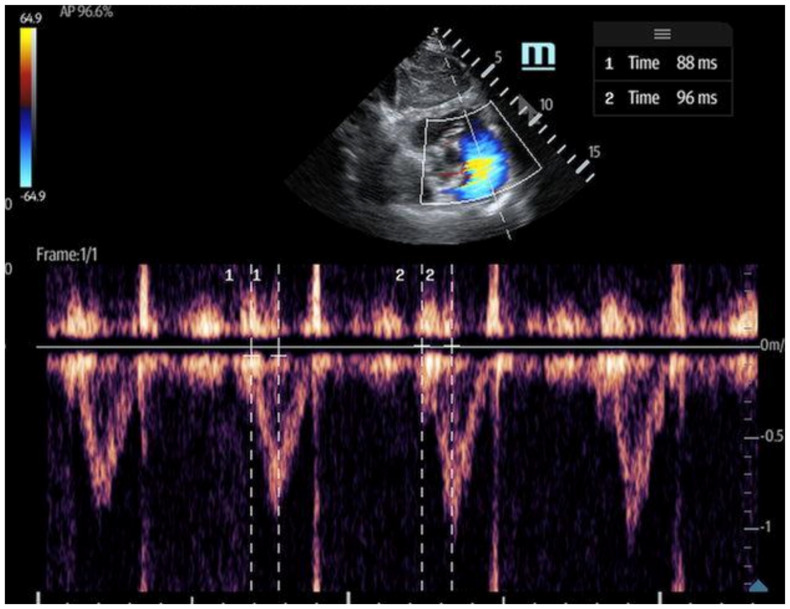
Pulmonary acceleration time (PAT) measured in the subcoastal short axis, and PW doppler activation measured as the interval between the onset of pulmonary flow and peak velocity.

Point-of-care ultrasound markers of RV afterload:

1. Tricuspid regurgitation (TR) jet velocity: Pulmonary artery (PA) pressures can be estimated using TR jet peak velocity (Figure 8a). The evidence suggests that in the right clinical context, a TR jet velocity greater than 2.8 m/s is an independent predictor of pulmonary hypertension, while a velocity > 3.4 m/s strongly indicates this condition [35,43,44]. However, this method has limitations as TR jet velocity may underestimate PA pressures in cases of severe TR and is unreliable when TR is insufficient for evaluation [43]. Additionally, this technique cannot differentiate pulmonary hypertension from other causes of RV outflow obstruction, such as pulmonary or RVOT stenosis [44].
2. McConnell’s sign: A POCUS finding defined as hypo or akinesis of the mid-RV free wall with preserved apical contraction on ultrasound is a widely recognized marker of right heart strain, particularly in the setting of acute pulmonary embolism (PE) [32,35]. In the original 1996 study describing McConnell’s sign, a sensitivity of 77% and a specificity of 94% were markers for acute PE, even after comparison with patients with other causes of RV dysfunction, such as pulmonary hypertension [45,46]. However, additional studies since then have cast doubt on the high specificity of McConnell’s sign, with one study showing equal prevalence of McConnell’s sign in patients with acute PE or RV myocardial infarction and another study demonstrating that patients diagnosed with acute PE with McConnell’s sign present had similar echocardiographic findings as a comparison group of patients with chronic pulmonary hypertension [47,48]. In contrast, a recent meta-analysis still found a pooled sensitivity of 22% and specificity of 97% for McConnell’s sign in the setting of acute PE, although they noted that high suspicion for acute PE prompted POCUS use in the majority of the studies [49]. Its positive predictive value ranges between 57 and 71% [50]. These findings suggest that while McConnell’s sign may be consistent with right heart strain in acute PE, caution should be utilized regarding its diagnostic capabilities for PE. Accordingly, in 2010, the American Society of Echocardiography and American College of Emergency Physicians recommended against the sole use of POCUS and McConnell’s sign for diagnosing and treating presumed acute PE except in patients too unstable to undergo additional confirmatory testing such as CT imaging [51].
3. The “D-Sign”: This refers to the flattening of the interventricular septum with a leftward deviation into the left ventricle, causing the left ventricle to appear as a D-shaped structure, as seen in the parasternal short-axis view (Figure 8b). This finding suggests increased RV afterload and can be associated with pulmonary hypertension, acute PE, and RV failure [35]. Diastolic septal flattening indicates volume overload (e.g., severe tricuspid regurgitation or atrial septal defect), while systolic flattening points to pressure overload (e.g., pulmonary hypertension or acute PE). In volume overload, the D shape is most pronounced at end-diastole and resolves in systole, whereas in pressure overload, flattening persists throughout the cardiac cycle [36].
4. The 60/60 sign: This is an echocardiography sign that refers to the presence of both pulmonary acceleration time (PAT) ≤60 milliseconds (Figure 9) and tricuspid pressure gradient ≤60 mmHg as a marker for acute RV strain in acute PE, although it has poor sensitivity [52]. It is thought to be more advantageous than McConnell’s sign due to its objectivity and reproducibility [50]. The 60/60 sign has a sensitivity of 36% and a specificity of 94% when seen in combination with McConnell’s sign, emphasizing the utility of various ultrasound findings when assessed in conjunction with each other [50].

Although McConnell’s sign and the 60/60 sign demonstrate high specificity for acute PE, their sensitivity remains relatively low [45,48]. Notably, methodological differences across studies, including variable operator experience, patient selection, and reference standards, have contributed to inconsistent diagnostic performance. Importantly, both signs should be reserved for high-risk PE patients (e.g., those with hemodynamic instability or cardiac arrest), where rapid bedside assessment is vital to minimize the risk of misdiagnosis or overdiagnosis in lower-risk settings.

## 4. Left Ventricular Assessment and Integration of Ultrasound

### 4.1. Left Ventricular Preload

Left ventricular (LV) preload is influenced by multiple factors, many of which overlap with those determining RV preload. However, an additional key method of assessing LV preload independent of RV function is an ultrasound of the lung to determine extravascular lung water (EVLW).

**Figure 10:**
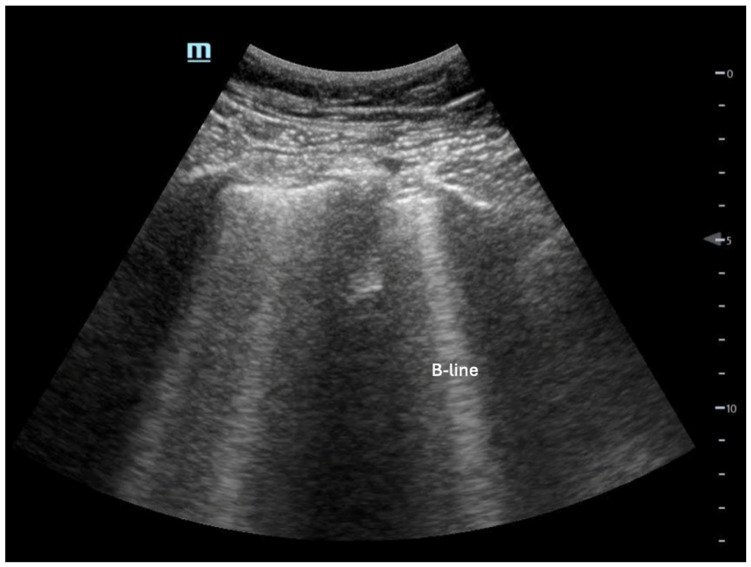
Example of B-lines, which are discrete vertical hyperechoic artifacts that originate at the pleural line and extend to the bottom of the screen.

Extravascular lung water: This is indicative of LV congestion, which can be detected with high sensitivity by identifying B-lines on lung ultrasound [53] (Figure 10). The presence of three or more B-lines in an intercostal space across multiple lung fields suggests possible cardiogenic pulmonary edema, though alternative causes, such as acute respiratory distress syndrome (ARDS) or interstitial lung disease, should also be considered. Pathologic B-lines are vertical, hyperechoic artifacts extending from the pleural line to the deep lung parenchyma, disrupting normal A-lines. Distinguishing true B-lines from comet-tail artifacts is essential, as comet-tails also originate from the pleural line but do not extend as deeply. Additionally, isolated B-lines (1–2 per lung field) may be a normal finding and should not be overinterpreted. A systematic lung ultrasound assessment, combined with clinical correlation, is crucial for differentiating cardiogenic from non-cardiogenic pulmonary edema.

### 4.2. Left Ventricular Function

Cardiac output (CO) and stroke volume (SV) are fundamental to managing circulatory failure. Stroke volume, the amount of blood ejected per heartbeat, provides critical insight into cardiac function, guiding diagnosis, influencing management decisions, and allowing for the trending of therapeutic interventions. While traditionally measured through invasive monitoring in ICU or operating room settings, point-of-care ultrasound (POCUS) has made CO and SV assessment more accessible across various clinical environments.

While ultrasound-derived CO has been compared to pulmonary artery catheterization, studies suggest that the two methods may not be entirely interchangeable. However, significant agreement has been demonstrated, particularly in mechanically ventilated patients [12]. The ability to non-invasively monitor CO and SV at the bedside enables the dynamic assessment of volume responsiveness and guides the administration of fluids, inotropes, and vasopressors.

#### 4.2.1. Point-of-Care Ultrasound Assessment of Left Ventricular Function

**Figure 11:**
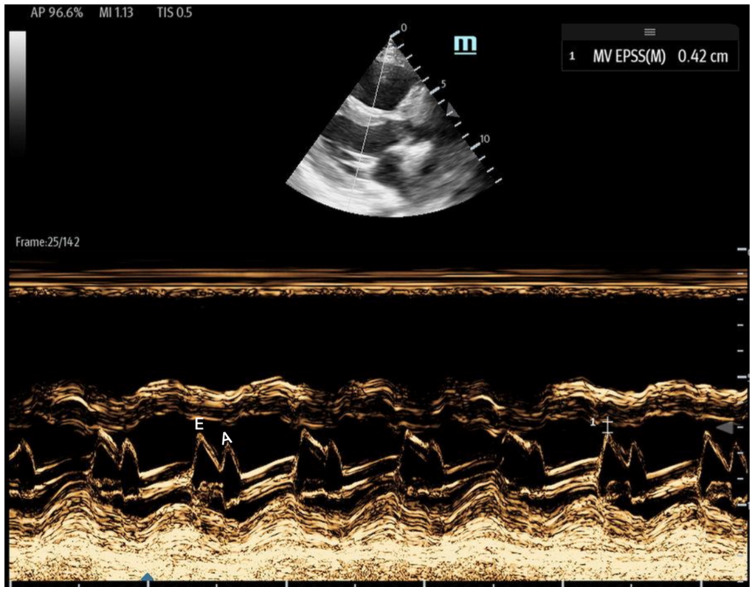
The parasternal long-axis view with the M-mode shows the EPSS measurement, which is the minimal distance between the **E** wave (initial and maximal opening of the mitral for the passive filling of LV) and the septum. The E wave is followed by the **A** wave, which is smaller and corresponds to left atrial contraction.

Left ventricular ejection fraction (LVEF) is a key marker of systolic function and can be estimated using several echocardiographic techniques:

1. 1E-point Septal Separation (EPSS): Obtained in the parasternal long-axis (PLAX) view, EPSS quantifies the distance between the anterior mitral valve leaflet tip and the interventricular septum (Figure 11). An EPSS greater than 1 cm may indicate a reduced LVEF of less than 40%, with a sensitivity of 69% and specificity of 91% [54]. This method requires only brief bedside training [55]. However, EPSS may not accurately reflect cardiac function in certain patients. In those with valvular pathologies (e.g., mitral stenosis/prosthesis or aortic regurgitation), abnormal valve or leaflet movement can lead to falsely high EPSS measurements despite normal LVEF. Septal hypertrophy may also underestimate EPSS. Lastly, in atrial fibrillation, the lack of coordinated atrial contractions necessitates multiple measurements for reliable assessments [56].
2. Fractional Shortening (FS): Obtained in the PLAX view, FS measures the left ventricular (LV) end-systolic and end-diastolic diameters. It evaluates the percentage change in LV diameter between diastole and systole, which is calculated as:
  (4) $$FS\left(\%\right)=\frac{LVEDD-LVESD}{LVEDD}\times 100$$
  FS serves as an estimate of how effectively the LV contracts and can be used as a surrogate for LVEF. However, it may be falsely low in areas of wall motion abnormalities and is highly influenced by preload and afterload. Additionally, FS has been shown to underestimate LVEF in patients with concentric left ventricular hypertrophy [57,58].

#### 4.2.2. Left Ventricular Velocity–Time Integral (LVOT-VTI)

In hemodynamically unstable patients, the LVEF may not reliably reflect SV in hyperdynamic conditions, and a high LVEF can be accompanied by reduced SV, whereas patients with a severely reduced LVEF may still have normal SV [59]. SV offers real-time data on the effectiveness of interventions like inotropes and is crucial for calculating CO, making it one of the most accurate echocardiographic markers for estimating SV and CO [60]. Additionally, SV is a marker of preload sensitivity, which will be discussed further below.

To estimate SV and CO, ultrasound requires two key measurements: the LVOT diameter and the LVOT-VTI. Cardiac output can also be indexed to body surface area to calculate the cardiac index (CI), further enhancing the hemodynamic assessment.

**Figure 12:**
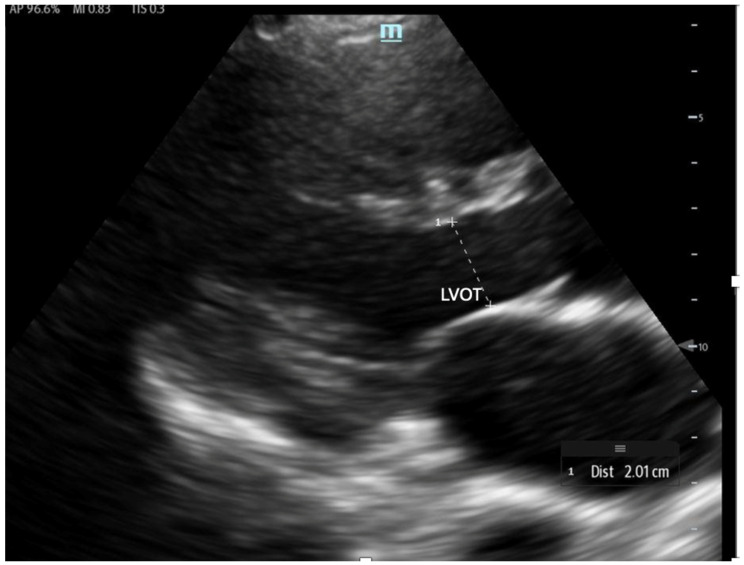
The LVOT diameter measured below the opening of the aortic valve in a zoomed-in parasternal long axis to be incorporated into the LVOT area formula.

The LVOT diameter is measured in the PLAX view, focusing on the LVOT and aortic valve when maximally open during mid-systole (Figure 12). The measurement is taken at the base of the aortic valve, and the area is calculated using the formula:

(5) LVOT Area=3.14×(radius of LVOT)²

**Figure 13:**
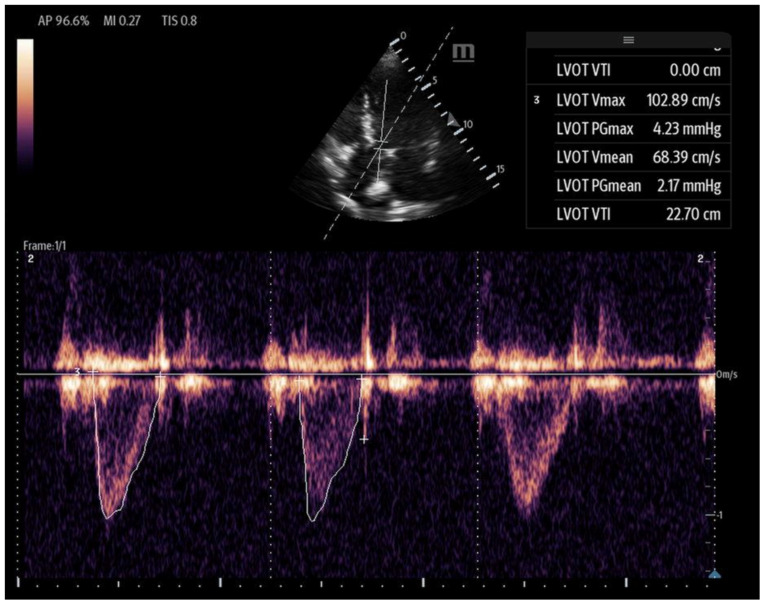
Left ventricular VTI calculated with PW Doppler with Doppler gate at LVOT.

The VTI is measured in the apical five-chamber view using pulse-wave Doppler, with the sample volume placed at the center of the LVOT to trace the velocity curve (Figure 13). The VTI, measured in centimeters and averaged over several beats to account for beat-to-beat variability, reflects the distance that blood is propelled forward from the LVOT to the aorta with each contraction. When used in conjunction with blood pressure and lactate levels, the LVOT-VTI can help guide shock etiology as well. A low VTI with hypotension and elevated lactate suggests low-output shock (e.g., cardiogenic), and a high VTI with hypotension and elevated lactate may suggest distributive shock (e.g., early sepsis). The normal range is between 17 and 23 cm, and it serves as a surrogate for stroke volume. When used in conjunction with the clinical picture, the VTI can also serve as a valuable tool for longitudinal assessment and is shown to decrease total time on vasoactive agents [61].

Left ventricular diastolic function:

Assessing left ventricular (LV) relaxation offers key insights into left-sided filling pressures, which can guide fluid and diuretic management based on the patient’s volume status. This evaluation also aids in distinguishing between pulmonary edema caused by elevated left atrial pressure (LAP) or primary lung disease. Imaging mitral inflow and myocardial tissue velocities allows the identification of abnormal relaxation patterns, with two commonly used point-of-care ultrasound measurements—mitral inflow and tissue Doppler—demonstrating 90% accuracy in diagnosing diastolic dysfunction in ICU patients [62].

**Figure 14:**
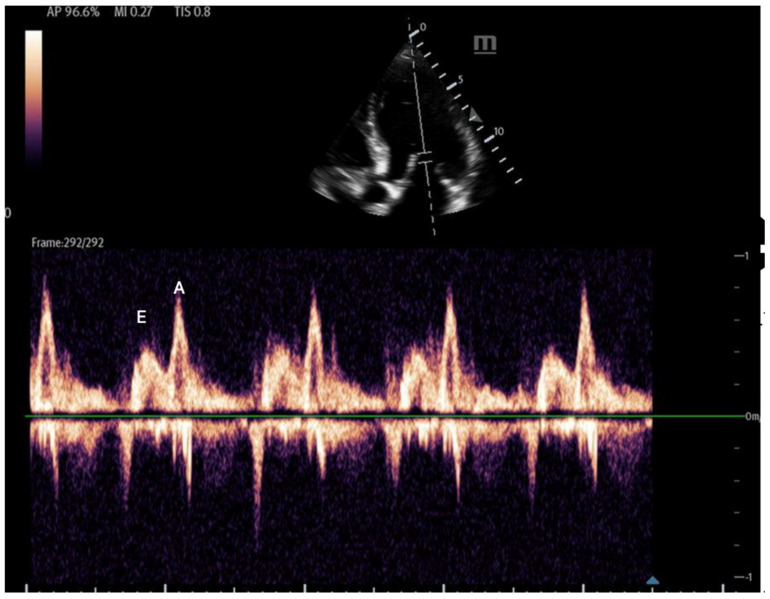
Mitral inflow with PW Doppler gate at mitral valve tip (E and A waves).

**Figure 15:**
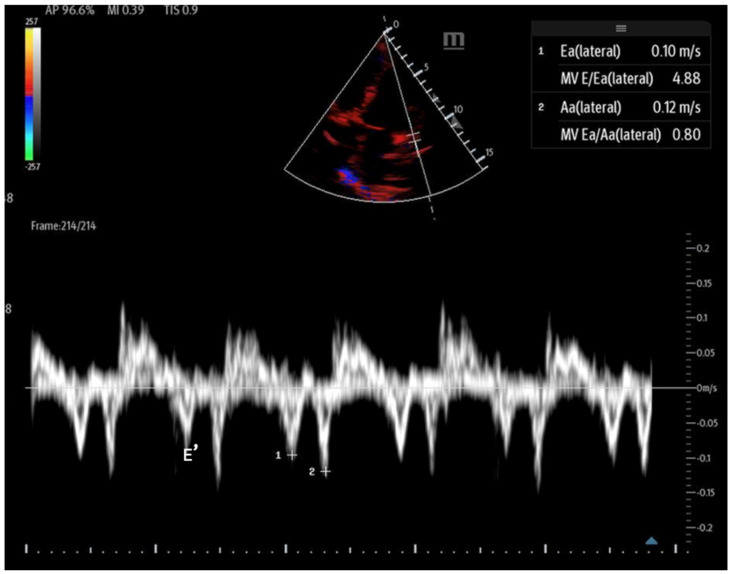
Tissue Doppler at the lateral septal annulus to record the E′ wave (labeled).

- Mitral inflow: Assessed via pulsed-wave (PW) Doppler at the mitral valve tips in the apical four-chamber view, this measures the blood flow velocities entering the left ventricle as an upward deflection of the PW Doppler signal. The resultant E and A waves are then evaluated for their ratio (Figure 14).
- Tissue Doppler: Measures myocardial movement during diastole using tissue Doppler at the septal annulus. The E/e′ ratio is calculated from the recorded e′ wave (Figure 15).

When abnormal, these values can indicate diastolic dysfunction and its severity (Table 3). However, certain factors, such as mitral annular calcification, basal wall motion abnormalities, mitral stenosis, and significant mitral regurgitation, can affect mitral inflow velocities and invalidate measurements. Additionally, tachycardia may cause the fusion of the E and A waves, and atrial fibrillation can lead to variable RR intervals, further complicating accurate assessment [63]. It is important that while these assessments can be performed with bedside POCUS, accurately interpreting advanced diastolic parameters often exceeds the scope of non-cardiologist operators and may necessitate formal transthoracic echocardiography.

### 4.3. Left Ventricular Afterload

Assessing LV afterload includes evaluating dynamic interventricular gradients, such as LV outflow tract obstruction (LVOTO), which can significantly impact hemodynamic management. The presence of LVOTO may guide the use of medications or fluids to optimize preload and afterload while avoiding inotropic agents that could exacerbate the obstruction. Continuous wave Doppler applied across the LV outflow tract reveals a characteristic late-peaking, “dagger-shaped” waveform, indicating hemodynamically significant LVOTO. This can be differentiated from aortic stenosis, which typically presents with a more symmetric waveform. A peak outflow gradient greater than 30 mmHg often suggests a significant dynamic LVOTO. Causes of LVOTO include the systolic anterior motion of the mitral valve, infiltrative cardiac disease, and hypertrophic cardiomyopathy [65]. Hypovolemia, dehydration, and shock can exacerbate LVOTO by reducing preload and promoting SAM, even without structural heart disease. This load-dependent phenomenon is well recognized in critically ill patients and often resolves with volume resuscitation and reduced inotropic stimulation [66].

While bedside ultrasound provides invaluable real-time hemodynamic data, it has limitations. One notable challenge is the difficulty in visualizing the thoracic aorta, which may restrict the assessment of acute aortic syndromes. Nonetheless, when combined with the clinical context and other echocardiographic findings, POCUS remains an essential tool for guiding circulatory management in a wide range of settings.

## 5. Detecting Fluid Responsiveness Using POCUS

Preload sensitivity or fluid responsiveness is critical in shock management to avoid the risks of over-resuscitation [67]. The focus has shifted from simply increasing oxygen delivery through fluid to managing fluid tolerance, with an emphasis on minimizing organ congestion and dysfunction [68]. Point-of-care ultrasound (POCUS) is especially valuable in ambiguous clinical settings where the volume status is unclear. Dynamic measures provided by POCUS, rather than single data points, are key to assessing preload sensitivity.

Echocardiography with POCUS at the bedside is a non-invasive, validated approach to answering these questions. Key measures include the left ventricular outflow tract (LVOT) diameter and LVOT velocity time integral (VTI), which can be used to calculate cardiac output (CO) and stroke volume (SV):

(6) $$LV\;Stroke\;volume=LVOT\;area\;\left(cm^{2}\right)\times LVOT\;VTI\;(cm)$$

Various methods assess fluid responsiveness during echocardiographic testing, including fluid challenge response, passive leg raise, pulse pressure variability, stroke volume variation (SVV), and the end-expiratory occlusion test [69]. For instance, a respiro-phasic variation in stroke volume, particularly during mechanical ventilation, helps assess preload sensitivity. For accurate interpretation, dynamic measures, like SVV, require patients to be on controlled mechanical ventilation with a tidal volume of ≥8 mL/kg, an ideal body weight, the absence of spontaneous breathing activity, and in regular sinus rhythm [70].

Another method is the passive leg raise test, which measures the LVOT VTI in the supine position and after raising the legs. A significant increase in the VTI or the stroke volume index indicates preload sensitivity, with a cutoff of 14% suggesting fluid responsiveness [71].

In comparison to dynamic measures that rely on cardiopulmonary interactions, ultrasound parameters, especially the LVOT-VTI, have been shown to predict fluid responsiveness, even in patients with low tidal volume or spontaneous breathing [72,73].

Lastly, the ultrasonographic measurement of corrected carotid flow time (cCFT), assessed using pulse-wave Doppler, correlates with stroke volume and can help assess preload responsiveness. Some small studies have demonstrated high specificity (96%) in predicting fluid responsiveness using a passive leg raise [74]. When corrected for heart rate, a normal CFT value of about 300 ms corresponds to a normal stroke volume [67].

## 6. Transesophageal Echocardiography (TEE)

Point-of-care transesophageal echocardiography (TEE) is a highly valuable tool in assessing hemodynamics and guiding management, particularly when transthoracic echocardiography (TTE) views are limited due to anatomical or clinical conditions. It offers high-quality images that can have superior diagnostic capability in settings like hemodynamic instability, predicting fluid responsiveness, and evaluating cardiac function [75,76]. TEE is beneficial across various clinical settings, including hemodynamic instability, cardiac arrest, the perioperative period, and pulmonary distress via transesophageal lung ultrasound (TELUS). It is also useful for prone-positioned patients, such as those with ARDS. While TEE is slightly more invasive than TTE, it carries minimal risk, though it is contraindicated in patients with significant esophageal pathology (e.g., strictures, tumors, and bleeding varices), perforated viscus, or in situations where sedation may be hemodynamically taxing restricted neck mobility or severe thrombocytopenia or neutropenia are present. Care should be taken to prevent endotracheal tube dislodgement in intubated patients.

Standard TEE views include the midesophageal window (four-chamber, long-axis, and bicaval views) and the trans-gastric short-axis view. These allow for the evaluation of cardiac output (CO), left ventricular (LV) filling pressures, mitral regurgitation, tamponade physiology, and acute aortic syndromes, as well as right and left ventricular dysfunction. Additionally, TEE provides excellent visualization of vessels, including the superior vena cava (SVC), which can be used to calculate the SVC collapsibility index. An SVC collapse of 21% or more can serve as a dynamic indicator of fluid responsiveness, with 81–84% specificity [73,77].

TEE is also valuable in cardiac arrest to determine the etiology of the arrest and guide resuscitation efforts. Its advantages of focused TEE over TTE include minimizing interruptions and facilitating image acquisition more easily in cardiac arrest patients [78]. Moreover, TEE can assist with procedural guidance in various scenarios, such as ECMO cannulation, intra-aortic balloon pump (IABP) placement, right atrial (RA) catheter insertion, and pacemaker positioning. However, the routine use of TEE in cardiac arrest has not been universally supported by systematic reviews, mainly due to methodological variability in studies [79].

Despite its utility, challenges to the widespread use of POCUS TEE include a lack of standardized training, the cost of equipment, and potential complications, such as oropharyngeal and gastrointestinal injury, aspiration, or transient hypoxia. Understanding the risk of the procedure and incorporating mitigation strategies such as pre-procedure screening and airway protection is essential for safe and effective use. Further research is necessary to better define the impact of TEE on clinical outcomes in hemodynamic instability and cardiac arrest.

## 7. Ultrasound Evaluation of Indwelling Hemodynamic Monitors and Assist Devices

Indwelling devices such as the pulmonary artery catheter (PAC) are commonly used in critical care to monitor hemodynamics. Ultrasound serves as an adjunct to confirm catheter placement and functionality. For PAC evaluation, parasternal short-axis and subcostal views can identify the proper placement of the catheter tip, ensuring it is not improperly wedged or causing retrograde migration into the right ventricle, which can lead to arrhythmias. Echocardiography can also detect mild tricuspid and pulmonic regurgitation due to catheter placement.

Echocardiography is also essential in the evaluation of axial flow pumps, like the Impella™ device, used in mechanical circulatory support. Correct positioning is critical for optimal device function and to prevent suction alarms. The device should be positioned 4–4.5 cm from the aortic valve for LV support devices or within the inferior vena cava for RV support devices, as visualized in the parasternal long-axis view.

## 8. Discussion

Ultrasound is a robust, non-invasive, and non-ionizing modality that provides valuable bedside insights into physiology, particularly from a macrocirculatory perspective. This review has outlined various methods for assessing hemodynamics, beginning with right ventricular (RV) function, followed by left ventricular (LV) function. While clinicians may focus on one side at a time, it is common practice to assess both RV and LV function simultaneously, as they are interdependent in maintaining overall cardiac performance. The tools discussed here can help clinicians narrow down a specific hemodynamic problem while simultaneously guiding resuscitative interventions for hemodynamically unstable patients during shock states (see Table 1).

The standard approach to hemodynamic assessment described in this review, if performed in sequence, provides a comprehensive understanding of a patient’s status. This includes evaluating volume status, volume responsiveness, and both systolic and diastolic cardiac function.

In addition to standalone use, POCUS can be effectively integrated with other hemodynamic monitoring modalities to enhance diagnostic accuracy. Techniques such as arterial pulse contour analysis, transpulmonary thermodilution, and bioimpedance monitoring offer complementary data on cardiac output, preload, and systemic vascular resistance. When used synergistically, POCUS enhances the interpretation of these parameters by providing direct structural dynamic insight into cardiac performance and filling status. This can support more individualized shock management strategies.

Despite its many advantages, ultrasound has several limitations that must be considered. First, it is highly operator-dependent. The accuracy of the findings is directly influenced by the operator’s experience and proficiency, which can lead to variability in interpretation. This highlights the need for structured training programs and institutional protocols to ensure quality and reproducibility. For instance, suboptimal imaging due to poor technique or inadequate probe positioning can compromise the quality of the assessment. Second, technical constraints, such as the patient’s body habitus, can pose challenges. In obese patients or those with challenging anatomical features, obtaining clear and reproducible images may be difficult. Additionally, the presence of gas-filled structures, such as in patients with lung disease, may interfere with acoustic transmission, limiting the effectiveness of ultrasound in certain clinical situations.

The utility of POCUS may also be reduced in specific patient populations. In pregnant patients, physiological changes like increased blood volume and altered venous return can affect baseline measurements. In patients with severe pulmonary hypertension, complex right heart physiology can lead to the misinterpretation of volume status or cardiac function. These factors should be considered when applying POCUS findings in these contexts.

Furthermore, several common confounding factors may also impact the accuracy of hemodynamic assessments. For instance, positive-pressure ventilation alters intrathoracic pressure and venous return, which can influence measurements like IVC. Similarly, valvular heart diseases may distort Doppler measurements, while arrhythmias, particularly atrial fibrillation, can introduce beat-to-beat variability that reduces reliability. Awareness of these limitations is essential for appropriate interpretation and use in clinical decision-making.

Ultimately, these limitations highlight the importance of experienced operators and an understanding of the technical nuances involved. POCUS findings should be interpreted within the clinical context and integrated with other diagnostic information to guide management. Despite these challenges, when performed correctly, ultrasound remains a powerful tool in dynamic hemodynamic evaluation and can significantly enhance clinical decision-making in critical care.

## 9. Future Directions

Artificial intelligence (AI) is transforming various areas of medicine, and ultrasound-guided hemodynamic assessment is no exception. Feasibility studies have demonstrated that AI can significantly enhance the abilities of less experienced operators by improving the accuracy of measurements and reducing inter-operator variability. For example, AI algorithms can assist in evaluating cardiac output more precisely, making it easier for less experienced operators to produce reliable results [80]. As AI technologies continue to evolve, we can expect even greater refinement in the automation of echocardiographic parameters, such as the auto-measurement of the inferior vena cava (IVC) and velocity–time integral (VTI). These advancements will not only increase the clinical utility of ultrasound but also ensure that such assessments can be applied to a wider range of clinical scenarios with greater consistency and accuracy.

In addition to technological advancements, there is a growing emphasis on increasing bedside education for the ultrasonographic assessment of hemodynamics. This education is crucial for the broader adoption of ultrasound in clinical practice. Many commercial and institutional training programs are now available, aiming to train clinicians, from medical students to experienced postgraduate trainees. However, to maximize the effectiveness of these programs, the standardization of training curricula is necessary. This would help reduce inter-operator variability, ensuring that clinicians at all levels can achieve reliable and reproducible results.

Furthermore, the development of portable and handheld ultrasound probes, which can be easily synchronized with personal electronic devices, is revolutionizing access to ultrasound technology. This innovation enhances patient evaluation across diverse settings, from emergency rooms to remote locations. In addition, emerging opportunities in teleassistance, teleconsultation, and telemonitoring are expanding the reach of POCUS, allowing real-time guidance and interpretation support across distances. The integration of AI into these settings further enhances image acquisition, interpretation accuracy, and decision-making support, potentially improving both diagnostic efficiency and access to expert-level assessment.

## 10. Conclusions

Point-of-care ultrasound offers a powerful, non-invasive approach to assessing hemodynamics, providing valuable insights into volume status, fluid responsiveness, and cardiac function. When incorporated into clinical practice within appropriate clinical contexts, it can enhance decision-making in the treatment of complex critically ill patients.

## Figures and Tables

**Table 1:** Summary of POCUS findings across different shock states, which highlights potential sonographic clues for determining shock etiology [7].

POCUS Exam	Hypovolemic	Cardiogenic	Obstructive	Distributive
Heart	Hyperdynamic left ventricle (LV)	Dilated chambers or reduced LV/RV function	Pulmonary embolism (PE): dilated, strained RV, with a D-shaped septum and positive McConell’s sign. Tamponade: pericardial effusion with RA systolic and RV diastolic collapse	Hyperdynamic LV in early sepsis and hypodynamic in late sepsis
Lung	A-Line pattern (normal lung)	B-lines and/or pleural effusion	PE: small pleural effusion or pulmonary infarcts (subpleural consolidations) Pneumothorax: absent lung sliding	Pneumonia: consolidation and/or focal B-lines, pleural effusion
IVC	Small and collapsible	Distended, non-collapsible	Distended, non-collapsible	Normal or collapsed
Others	Abdominal exam may show abdominal aortic aneurysm, aortic dissection, or intra-abdominal hemorrhage. Vascular exam may reveal deep vein thrombosis (DVT) or collapsed vessels	Abdomen exam may show peritoneal fluid	Vascular exam may show DVT	An abdomen exam could show peritoneal fluid. Musculoskeletal ultrasound may detect a focal abscess as a fluid collection

**Table 2:** Summary of right ventricular contractility parameters: Sensitivity and specificity values vary by study population and methodology; these reflect pooled or representative values from cited literature.

Parameter	Normal Value	Sensitivity	Specificity	Reference
TAPSE	≥16 mm	74%	81%	[33]
RVOT-VTI	≥12 cm	84%	78%	[34,35,36]
S′	≥10 cm/s	90%	85%	[37]

**Table 3:** Grading for diastolic dysfunction based on mitral inflow and tissue Doppler [64].

Normal	Grade 1 (Impaired Relaxation)	Grade 2 (Pseudo Normal)	Grade 3 (Restrictive)
E/A ≥ 0.8 e′ ≥ 8 cm/s E/e′ < 8	E/A < 0.8 e′ < 8 cm/s E/e′ <8	E/A ≥ 0.8 e′ < 8 cm/s E/e′ 8–15	E/A ≥ 2 e′ < 8 cm/s E/e′ > 15
